# Vascular-adhesion protein 1 in giant cell arteritis and polymyalgia rheumatica

**DOI:** 10.3389/fmed.2024.1448157

**Published:** 2024-08-14

**Authors:** Simon M. Petzinna, Claus-Jürgen Bauer, Valentin S. Schäfer

**Affiliations:** Department of Rheumatology and Clinical Immunology, Clinic of Internal Medicine III, University Hospital of Bonn, Bonn, Germany

**Keywords:** giant cell arteritis (GCA), polymyalgia rheumatica (PMR), large vessel vasculitides (LVV), immunology & inflammation, vasculitis

## Abstract

Vascular adhesion protein-1 (VAP-1) is a type 2 transmembrane sialoglycoprotein with oxidative deamination functionality, encoded by the amine oxidase copper-containing 3 (AOC3) gene. VAP-1 is widely expressed across various tissues, particularly in highly vascularized tissues and organs essential for lymphocyte circulation. In the vascular system, VAP-1 is predominantly found in vascular smooth muscle cells and endothelial cells, with higher expression levels in vascular smooth muscle cells. Under inflammatory conditions, VAP-1 rapidly translocates to the endothelial cell surface, facilitating leukocyte adhesion and migration through interactions with specific ligands, such as sialic acid-binding immunoglobulin-type lectins (Siglec)-9 on neutrophils and monocytes, and Siglec-10 on B cells, monocytes, and eosinophils. This interaction is crucial for leukocyte transmigration into inflamed tissues. Furthermore, VAP-1’s enzymatic activity generates hydrogen peroxide and advanced glycation end-products, contributing to cytotoxic damage and vascular inflammation. In this context, the soluble form of VAP-1 (sVAP-1), produced by matrix metalloproteinase cleavage from its membrane-bound counterpart, also significantly influences leukocyte migration. This review aims to elucidate the multifaceted pathophysiological roles of VAP-1 in vascular inflammation, particularly in giant cell arteritis (GCA) and associated polymyalgia rheumatica (PMR). By exploring its involvement in immune cell adhesion, migration, and its enzymatic contributions to oxidative stress and tissue damage, we investigate the importance of VAP-1 in GCA. Additionally, we discuss recent advancements in imaging techniques targeting VAP-1, such as [^68^Ga]Ga-DOTA-Siglec-9 PET/CT, which have provided new insights into VAP-1’s role in GCA and PMR. Overall, understanding VAP-1’s comprehensive roles could pave the way for improved strategies in managing these conditions.

## Introduction

Giant cell arteritis (GCA) is an immune-mediated vasculitis that affects large and medium-sized vessels, predominantly in individuals over 50 years of age. It is the most prevalent form of vasculitis in Western populations. GCA can lead to vascular changes and occlusion due to severe vascular inflammation, neoangiogenesis, and remodeling. Additionally, GCA is closely associated with polymyalgia rheumatica (PMR), which is characterized by inflammation in periarticular structures. PMR may precede, coincide with, or follow the onset of GCA. Thus, subclinical GCA can be detected in 22–23% of PMR patients (1, 2). However, in some studies, the incidence of large vessel vasculitis detected by positron emission tomography–computed tomography (PET/CT) in patients with PMR can reach up to 60%, particularly in those presenting with inflammatory low back pain, pelvic girdle pain, and diffuse lower limb pain (3, 4).

Despite advances in understanding the pathophysiology of GCA, the innate and adaptive immune mechanisms involved remain only partially understood. Initial hypotheses primarily attributed the immune response in GCA to TH1 cells, driven by the activation of the Janus kinase (JAK) and Signal Transducers and Activators of Transcription (STAT) signaling pathways (5). It has been demonstrated that IFN-γ plays a significant role in mediating chemotaxis through CXCL9, CXCL10, and CXCL11 in the arterial wall of GCA patients via the JAK-STAT1 pathway (6, 7). Moreover, functional polymorphisms of IFN-γ were associated with the development of severe ischemic complications of the disease (8). Recent findings, however, suggest that cytokines beyond the STAT signaling pathway may also significantly influence inflammation in GCA (5). Various mechanisms involving both TH1 and TH17 cells have been recognized, including the recruitment of T-cells within the vascular wall facilitated by vascular dendritic cells (9). These cells are responsible not only for chemotaxis and cytokine release but also for the differentiation of TH1/TH17 cells via vasculitogenic T-effector cells (9). A chemokine-mediated link involving IFN-γ, Interleukin (IL)-17, and IL-21 fosters an inflammatory environment (10, 11). Monocytes also significantly contribute to the differentiation of TH1 and TH17 cells via the production of cytokines such as IL-12p35 (promoting TH1) and IL-1β, IL-6, and IL-23p19 (promoting TH17) (12). Activated TH1/TH17 cells not only sustain the initial immune response by producing key cytokines (IFN-γ/IL-17) but also exacerbate inflammation by recruiting cytotoxic CD8 cells and monocytic precursor cells, which evolve into macrophages leading to vascular damage and remodeling (9).

The close connection between PMR and GCA has led to joint investigations into their disease mechanisms. In PMR, similar to GCA, Treg, TH1, and TH17-associated inflammatory processes, along with their key cytokines, are crucial (13–15). Moreover, IL-6, along with IL-1 and ICAM-1, is significantly implicated in the pathophysiology of PMR, influencing the likelihood of future relapses in patients (14–17). While ICAM-1 polymorphisms alone do not appear to be associated with disease severity in isolated PMR, the presence of homozygosity for both the HLA-DRB1*0401 allele and the 241 GG codon of ICAM-1 is significantly correlated with an increased risk of relapses in these patients (18). A major distinction in the immune response between PMR and GCA is the absence of a strong IFN-γ response in PMR (19).

While the understanding of immunological and pathophysiological aspects of GCA and PMR is evolving, significant gaps remain, particularly in linking immunological processes with disease manifestations. This emphasizes the need for a better understanding of these diseases.

## Structure and function of vascular-adhesion protein 1

Vascular adhesion protein-1 (VAP-1) is a type 2 transmembrane sialoglycoprotein, encoded by the amine oxidase copper-containing 3 (AOC3) gene. It forms a 180 kDa homodimer consisting of three distinct domains (D2-D4), capable of catalyzing oxidative deamination reactions (20–24). This enzymatic activity can be inhibited by semicarbazide, classifying VAP-1 within the semicarbazide-sensitive amine oxidase (SSAO) family (25). To clearly differentiate VAP-1-like SSAOs (topaquinone-containing amine oxidases) from other members of the SSAO family, they have been renamed as primary amine oxidases (26). Other SSAOs belong to the lysyl oxidase family, characterized by the presence of lysine tyrosyl quinone, rather than topaquinone, at their catalytic sites (26).

VAP-1 is expressed by various cell types, including vascular cells, pericytes on the outer surfaces of blood vessels, adipocytes, chondrocytes, follicular dendritic cells, and liver cells (26–30). Its expression is particularly prominent in tissues with high vascularization, such as blood vessels, muscle, cerebrovascular tissue, heart, liver, kidney, retina, intestine, lung, and adipose tissue. Moreover, VAP-1 is significantly expressed in organs involved in lymphocyte recirculation and homing, including the vessels of the spleen, thymic cortex, and lymph nodes (31–34).

In the vascular system, the expression of VAP-1 is predominantly observed in vascular smooth muscle cells (VSMC) and endothelial cells. VSMC, located in the medial layer of the vascular wall, exhibit higher expression and activity levels of VAP-1 compared to endothelial cells (26, 35, 36). VSMC are pivotal in producing the extracellular matrix, which is essential for the arterial wall’s resilience against blood circulation pressure and exhibit significant plasticity (37, 38). Under external stimuli, VSMC can migrate and proliferate from the medial to the intimal layer, contributing to intimal hyperplasia (38). VAP-1 in VSMC is specifically localized within the caveolae of the plasma membrane (39), yet the regulation of VAP-1 in these cells, as well as its physiological functions within them, remains less understood (26). Although VAP-1 in VSMC does not facilitate lymphocyte binding *in vitro* (39), it is implicated in critical processes such as vascular tone regulation, cell differentiation, and extracellular matrix organization (22, 40, 41). An increase in VAP-1 activity can generate reactive oxygen species, leading to VSMC death and potentially contributing to atherosclerosis (37, 38, 42).

**Figure 1:**
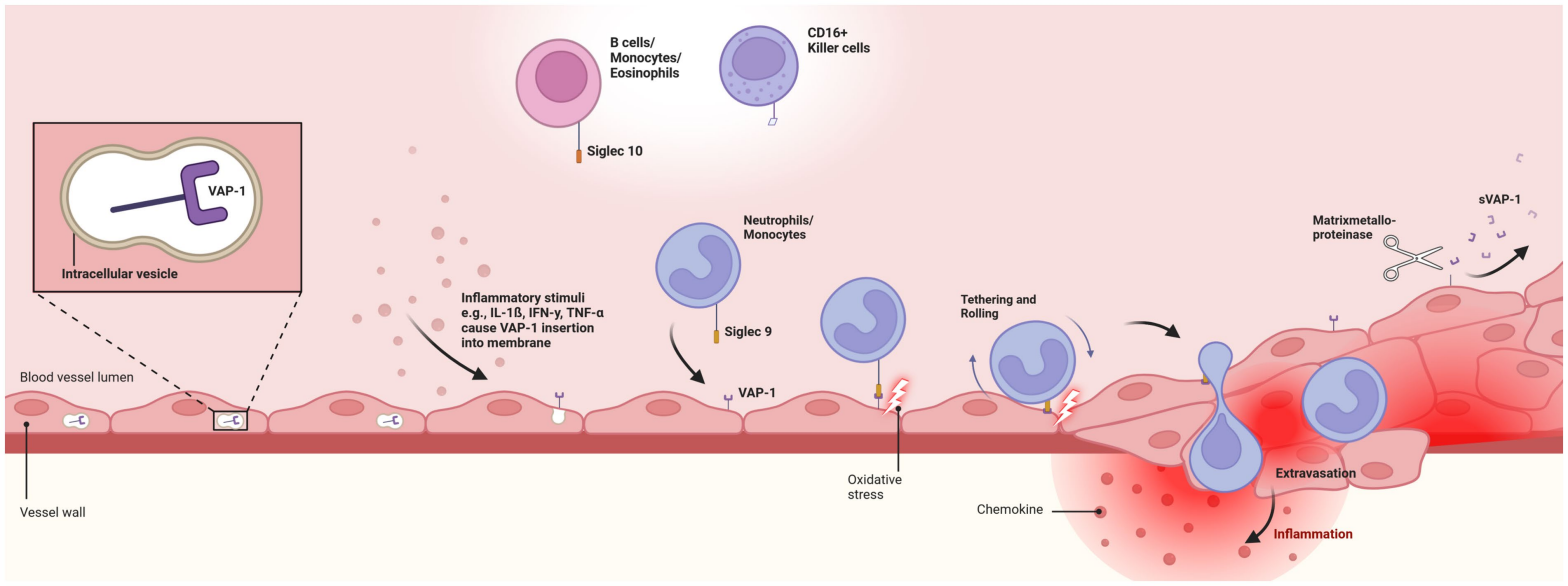
Pathophysiologal role of Vascular-adhesion protein 1. Depicts the endothelial translocation of membrane-bound vascular-adhesion protein 1 (VAP-1) from intracellular vesicles to the cell surface in response to inflammatory stimuli. Translocation facilitates the interaction of VAP-1 with circulating neutrophils and monocytes through the Siglec-9 ligand. This initiates oxidative deamination, leading to cytotoxic damage to endothelial cells and promoting an inflammatory response. Secretion of chemokines, activation of transcription factors, and expression of matrix metalloproteinases enhance leukocyte rolling, tethering, and migration. Finally, soluble VAP-1 (sVAP-1) is generated through the cleavage of membrane-bound VAP-1 by matrix metalloproteinases, releasing it into the circulation and significantly contributing to the monoamine oxidase activity in human blood. VAP-1, vascular-adhesion protein 1; sVAP-1, soluble vascular-adhesion protein 1. Created with BioRender.com.

Conversely, VAP-1 is present in all three types of endothelial cells, continuous, fenestrated, and sinusoidal (34). Its role varies across different tissue types and (patho-)physiological conditions. In specific endothelial cells, like liver sinusoidal endothelium and the specialized high endothelial venules in peripheral lymph nodes, VAP-1 is constitutively expressed on the cell surface (43, 44). In all other endothelial cells, VAP-1 resides within intracellular vesicles, absent from the cell surface. However, during inflammatory conditions, stimuli such as tumor necrosis factor-alpha, interferon-gamma, lipopolysaccharide, and interleukin-1ß trigger its rapid relocation to the surface of endothelial cells (26, 45–47) (Figure 1).

## Pathophysiological role of vascular-adhesion protein 1

VAP-1 plays a critical role in immune cell adhesion and migration, particularly facilitating the transmigration of leukocytes from the bloodstream into inflamed tissues. This process involves VAP-1’s dual function: its enzymatic activity catalyzes the oxidative deamination of primary amines (48), a key mechanism behind most of VAP-1’s pathophysiological effects, and its role as a membrane-bound endothelial adhesion molecule that supports enzyme-independent leukocyte binding (31). Under inflammatory conditions, VAP-1 therefore acts as an ectoenzyme with a catalytically active domain external to the cell membrane, amplifying its role in immune responses (38).

On endothelial cells, VAP-1 engages with leukocytes via specific ligands, including sialic acid-binding immunoglobulin-type lectins (Siglec)-9, predominantly found on neutrophils and monocytes, and Siglec-10, identified on B cells, monocytes, and eosinophils (48–51). Siglec-10 further acts as a substrate for VAP-1, a function not demonstrated for Siglec-9. Additionally, VAP-1 engages with CD16+ natural killer cells, although the precise mechanism of this interaction is not well understood (26, 45, 50–53).

Inflammatory stimuli lead to the rapid up-regulation of Siglec-9 and Siglec-10 on leukocytes, enhancing their interaction with endothelial VAP-1 (54, 55). This interaction fosters a transient adhesive bond, where primary amines on leukocytes serve as substrates for oxidative deamination by VAP-1’s catalytic site (50, 56). This two-step oxidative deamination process, converting methylamine to formaldehyde and aminoacetone to methylglyoxal, with subsequent production of hydrogen peroxide and ammonia contributing to advanced glycation end-products (AGE) formation and increased oxidative stress, inflicts cytotoxic damage on endothelial cells (56–58). This can result in vascular damage and potential vascular complications like atherosclerosis (26, 29, 31, 57, 59–61).

The generation of VAP-1-derived hydrogen peroxide, a powerful signaling molecule at low concentrations, plays an essential role in local inflammatory responses (26). The catalytic activity of VAP-1 induces the expression of various endothelial adhesion molecules, such as ICAM-1, MadCAM-1, E-selectin, and P-selectin, and promotes the secretion of the chemokine CXCL8 (44, 62–65). It also activates key transcription factors, facilitating the engagement of multiple signaling pathways, including PI3K, MAPK, and NF-κB, thereby fostering an inflammatory milieu beneficial to leukocyte extravasation (24, 56). This complex process encompasses leukocyte tethering and rolling along the endothelium, culminating in the extravasation cascade, essential for immune cell migration to sites of inflammation (24, 26, 27, 56, 66–68). Real-time imaging studies have underscored VAP-1’s facilitation of leukocyte slow rolling, firm adhesion, and subsequent migration within blood vessels, particularly at lymphoid tissues and inflamed sites (69). Notably, the interaction of various immune cells, including CD4+ helper T cells, T-regulatory cells, Th17 cells, CD8+ cytotoxic T cells, B lymphocytes, CD16+ monocytes, and granulocytes with high endothelial venules and flat-walled vessels, has been shown to be modulated, at least in part, by VAP-1 expression levels (34, 43, 52, 68, 70–76). This underscores VAP-1’s vital role in mediating immune surveillance and response, highlighting its importance in the immune system’s functionality.

While membrane-bound VAP-1 serves as a transmembrane glycoprotein within the vascular wall, soluble VAP-1 (sVAP-1) arises from the proteolytic cleavage of its membrane-bound form by matrix metalloproteinases, releasing it into circulation (26, 27, 59, 69). This allows VAP-1 to influence leukocyte migration in both transmembrane and soluble form, with sVAP-1 contributing significantly to the circulating monoamine oxidase activity in human blood (77). High concentrations of sVAP-1, often originating from high endothelial venules in lymphatic organs, play a crucial role in facilitating transendothelial migration of lymphocytes (78–80). In healthy individuals, sVAP-1 levels in the serum are typically low and stable, modulating the adhesive activity of its membrane-bound counterpart and enhancing leukocyte adhesion (26, 31, 79, 80).

Increased sVAP-1 expression is prevalent in various chronic inflammatory conditions (26, 81) with notable elevations in patients with type 1 diabetes and chronic liver diseases (80), as well as those suffering from skin inflammation (psoriasis), synovitis, active relapsing–remitting multiple sclerosis (RR-MS), and systemic lupus erythematosus (47, 79, 82–85). Furthermore, elevated serum VAP-1 activity is linked to vascular disorders including diabetes mellitus complications (86, 87), hypertension (56), congestive heart failure (88), multiple cerebral infarctions (89), Alzheimer’s disease (90), and atherosclerosis (91), where sVAP-1 levels correlate with intima-media thickness and the presence of carotid plaques (92, 93). Additionally, s VAP-1 concentrations can predict major adverse cardiovascular events and mortality (93, 94). These sVAP-1 increases are often associated with tissue-bound VAP-1 overexpression (60, 95). However, despite these associations, sVAP-1 can not be considered as a general inflammation marker due to the lack of consistent correlation with C-reactive protein levels (93).

The increasing recognition of VAP-1’s role in inflammation and its involvement in exacerbating local lesion formation has led to a growing body of research exploring its role across a spectrum of inflammatory conditions.

## Vascular-adhesion protein 1 in large vessel vasculitis and polymyalgia rheumatica

VAP-1’s pathophysiological role, along with the potential inflammatory and oxidative stress-inducing effects of its catalytic products, indicate its involvement in the pathogenesis of vascular inflammatory disorders. The specific localization of VAP-1 on the surface of blood vessel cells further corroborates its involvement in these diseases. This has prompted further investigations using imaging techniques targeting VAP-1 or its ligands. Recent advancements include the introduction of a novel inflammation-specific radiotracer, [^68^Ga]Ga-DOTA-Siglec-9, for evaluating inflammatory vascular diseases (50).

A recently presented studies demonstrated that [^68^Ga]Ga-DOTA-Siglec-9 PET/CT can detect vascular inflammation during relapses in GCA, revealing increased localized tracer uptake in regions such as the aorta and subclavian arteries (96, 97). Additionally, prednisolone treatment significantly influenced endothelial VAP-1 expression, suggesting a rapid, therapy-induced reduction of VAP-1. No significant association was found between C-reactive protein levels and tracer uptake, aligning with previous research in other diseases where no correlation could be found (93). Beyond its role as an endothelial adhesion molecule, elevated sVAP-1 has emerged as a potential biomarker for disease activity in GCA, with levels exceeding those in healthy controls (96). However, further studies are needed to confirm this finding. Comparably, in PMR, which is frequently associated with GCA, [^68^Ga]Ga-DOTA-Siglec-9 PET/CT has indicated involvement of VAP-1 (98). In a cohort of PMR patients, increased tracer uptake was observed in the shoulder and pelvic girdle regions, with a significant negative correlation between prednisolone intake and tracer uptake in the shoulder, further supporting the hypothesis that VAP-1 is rapidly eliminated following prednisolone exposure.

Currently, the precise mechanism of VAP-1 involvement in the pathogenesis and pathophysiology of GCA and PMR remains at least partly speculative. However, its role has been elucidated in other (autoimmune) diseases with vascular inflammation, suggesting potential parallels with the pathophysiology of GCA and PMR.

In granulomatosis with polyangiitis, VAP-1 is strongly expressed in the renal endothelium during active disease, indicating its potential role in glomerular endothelial cell injury and altered barrier function, thereby contributing to disease pathogenesis (99, 100).

Similarly, in neuronal *in vitro* endothelial cell models of the blood–brain barrier, a link has been identified between VAP-1 expression and endothelial cell activation. This relationship involves the altered release of pro-inflammatory and pro-angiogenic cytokines, along with subsequent activation of signaling cascades, that also have been shown to significantly contribute to pathogenesis of GCA. Thus, it has been shown that cells expressing human VAP-1 overproduce various cytokines related to inflammation in GCA [e.g., IL-6 (101, 102), IL-8 (103, 104), ICAM (102), VCAM (105, 106)] and trophic factors [e.g. VEGF (10, 107), NGF (108)] (109). The signaling pathways of VEGF and IL-8 are particularly implicated in activating the VEGFR2 molecular pathway, leading to increased endothelial permeability (109). Moreover, VEGF and VAP-1 (110) can be upregulated in response to hypoxia, suggesting that polymorphisms affecting VEGF may also impact processes involving VAP-1. These polymorphisms could potentially affect VAP-1 levels or activity, thereby modulating the extent and nature of inflammatory responses and the development of severe ischemic complications in GCA. In this context, VEGF-induced angiogenesis may contribute to GCA associated inflammation (111).

IL-6 signaling has also been explored as a potential driver of VAP-1-associated endothelial alterations in the blood–brain barrier model, with the STAT3 pathway, which is known to significantly contribute to the pathogenesis of GCA (5, 102), being notably more activated in endothelial cells expressing VAP-1. The significance of the IL-6-activated STAT3 pathway in these alterations was further demonstrated by the application of an IL-6 blocking antibody, which negated the permeability changes induced by VAP-1 conditioned media in wild-type cells (109). This may help explain the successful application of the Interleukin 6 receptor inhibitor Tocilizumab (112).

VAP-1 may also be a potential explanation for the successful treatment of GCA with methotrexate, which has shown efficacy in GCA (113) and PMR (114) and is currently under investigation for remission maintenance therapy in GCA (115). Studies in tumor necrosis factor-α-treated human umbilical vein endothelial cell lines have shown that methotrexate can downregulate pro-inflammatory genes, including VAP-1, highlighting endothelium-protective and anti-inflammatory effects of methotrexat (116).

Furthermore, VAP-1 has been recognized as significant in cerebral ischemic processes, offering a potential explanation for GCA-associated ischemic complications. In animal models with intracerebral hemorrhage-induced brain damage, VAP-1 inhibition downregulated the adhesion molecule ICAM-1 and diminished the infiltration of systemic immune cells, particularly neutrophils, to the injury site (117). This reduction in immune cell accumulation was accompanied by decreased pro-inflammatory cytokines, including TNF-α and MCP-1, and reduced activation of microglia/macrophages. Consequently, inhibiting VAP-1 curtailed the local inflammatory process, potentially reducing cerebral edema and enhancing neurobehavioral functions (117). Finally, in diabetic vascular complications, enhanced interactions between endothelial cells and lymphocytes mediated by VAP-1 (118) and the subsequent transmigration and recruitment of endothelial inflammatory mediators, are central to the activation and progression of various inflammatory pathways (31). Formaldehyde, methylglyoxal, and advanced glycation end-products may contribute to these complications in diabetes (63, 119).

In conclusion, VAP-1 has emerged as a pivotal factor in the pathogenesis and pathophysiology of various vascular inflammatory disorders, including GCA and PMR. The introduction of [68Ga]Ga-DOTA-Siglec-9 PET/CT has provided valuable insights, demonstrating VAP-1’s role in detecting vascular inflammation during GCA relapses and PMR diagnosis, while also highlighting the significant influence of prednisolone treatment on VAP-1 expression. Elevated VAP-1 levels further underscore its potential as a biomarker for disease activity in GCA, although additional studies are necessary to confirm these findings. Overall, the new understanding of VAP-1’s role in GCA and PMR underscores the necessity for continued research to further elucidate its mechanisms, paving the way for improved disease management of these conditions.
